# Olaratumab: A New Strategy in the Treatment of Advanced Soft-Tissue Sarcoma

**Published:** 2018-03-01

**Authors:** Donald C. Moore, Lesli A. Lavery

**Affiliations:** Levine Cancer Institute, Atrium Health, Rock Hill, South Carolina; and Levine Cancer Institute, Atrium Health, Charlotte, North Carolina

## Abstract

Olaratumab is a monoclonal antibody that recently received accelerated approval for the treatment of advanced soft-tissue sarcomas in combination with doxorubicin for a histologic subtype in which anthracycline-containing regimens is appropriate and disease is not amenable to curative surgery or radiotherapy. It inhibits platelet-derived growth factor receptor alpha, leading to the inhibition of tumor cell proliferation, angiogenesis, and metastasis. In a phase II clinical trial, olaratumab in combination with doxorubicin met its predefined primary endpoint of improving progression-free survival and secondary endpoint of overall survival compared to doxorubicin monotherapy in patients with advanced soft-tissue sarcoma. Common adverse events associated with the combination of olaratumab and doxorubicin include nausea, mucositis, neutropenia, and infusion-related reactions.

Sarcomas represent a heterogeneous group of mesenchymal malignancies that may arise in the soft tissue or bone (Skubitz et al., 2007). Soft-tissue sarcomas (STS) comprise less than 1% of all cancer diagnoses. About 80% of sarcoma cases in adults are STS (Siegel, Miller, & Jemal, 2018). It is estimated that there will be approximately 13,000 newly diagnosed cases of STS in the United States in 2018; there is estimated to be approximately 5,000 deaths due to STS in 2018 (Siegel et al., 2018). Prior radiation therapy is one of the most important risk factors for the development of this malignancy (Zahm & Fraumeni, 1997).

There have been more than 50 different histologic subtypes of STS identified, and differences exist in their behaviors, prognosis, and approach to treatment (Skubitz & D’Amado, 2007). Some of the most common subtypes of STS include leiomyosarcoma, liposarcoma, angiosarcoma, and gastrointestinal stromal tumors (GIST). The treatment landscape for GIST has expanded greatly in recent years with the introduction of tyrosine kinase inhibitors that target platelet-derived growth factor receptor (PDGFR). The use of these agents in this setting has led to improved outcomes and survival in this patient population.

Unfortunately, non-GIST STS has not experienced the same therapeutic advancements. For the past several decades, the first-line treatment of STS in adults has been generally limited to doxorubicin either as monotherapy or in combination with ifosfamide (Judson et al., 2014). Many of the clinical trials evaluating single-agent doxorubicin have yielded response rates between 10% to 25% (Borden, Amato, Edmonson, Ritch, & Shiraki, 1990; Edmonson et al., 1993; Judson et al., 2001; Mouridsen et al., 1987). While multidrug regimens for the first-line treatment of STS have the potential of improved response rates compared to doxorubicin monotherapy, there is also a potential for more toxicity (Judson et al., 2014).

In October 2016, the US Food and Drug Administration (FDA) granted accelerated approval to olaratumab (Lartruvo) for the treatment of adult patients with STS with a histologic subtype for which an anthracycline-containing regimen is appropriate and which is not amenable to curative treatment with radiotherapy or surgery (FDA, 2016). Olaratumab is the first monoclonal antibody FDA approved for the treatment of STS and is to be administered in combination with doxorubicin.

## PHARMACOLOGY AND PHARMACOKINETICS

Platelet-derived growth factor receptor alpha (PDGFR-α) is a tyrosine kinase receptor that is expressed on connective tissue cells (Heldin & Westermark, 1999). The activation of PDGFR-α leads to the stimulation of cellular growth. The overactivation of PDGFR-α may be implicated in the development of malignancies, in particular that of sarcoma (Ostman & Heldin, 2001). The activation of PDGFR-α can lead to tumor cell proliferation, metastasis, and maintenance of the tumor microenvironment (Henriksen et al., 1993, Sulzbacher, Birner, Träxler, Marberger, & Haitel, 2003). Olaratumab is a recombinant, fully human immunoglobulin G1 (IgG1) monoclonal antibody that targets and inhibits PDGFR-α (Eli Lilly and Company, 2017). Olaratumab interacts with PDGFR-α by preventing the binding of the ligands PDGF-AA and PDGF-BB to the receptor (Loizos et al., 2005). This interaction blocks the tyrosine kinase activity of PDGFR-α, which in turn shuts off the downstream signaling cascades that promote tumor cell proliferation, angiogenesis, and metastasis.

Mo and colleagues (2017) evaluated the pharmacokinetic profile of olaratumab as a single agent or in combination with chemotherapy. Patients included in this study had advanced solid tumor malignancies including non–small cell lung cancer, STS, GIST, and glioblastoma multiforme. The pharmacokinetics (PKs) of olaratumab 15 or 20 mg/kg were best described by a two-compartment PK model with linear clearance. This suggests full target saturation at the dose levels tested. Linear clearance was significantly affected by the patient’s body weight and tumor size, whereas central volume of distribution was only affected by body weight. Olaratumab elimination half-life was found to be approximately 11 days, which corresponds to a time of steady state of approximately 50 days. There was no difference in the PKs of olaratumab between patients who received single-agent olaratumab or in combination with chemotherapy, supporting the use of olaratumab in combination with chemotherapeutic agents without the need for dose adjustments.

## CLINICAL EFFICACY

Tap and colleagues (2016) conducted a multicenter, randomized phase Ib/II study to evaluate the safety and efficacy of olaratumab in combination with doxorubicin in patients with STS. Olaratumab plus doxorubicin was compared to doxorubicin monotherapy in adult patients with unresectable or metastatic STS who had not been previously treated with an anthracycline. Patients received a maximum of 8 cycles of doxorubicin 75 mg/m² on day 1 of a 21-day cycle; patients in the combination group also received olaratumab 15 mg/kg on days 1 and 8 until disease progression. The primary endpoint was progression-free survival (PFS). Secondary endpoints included overall survival, objective response rate, and safety. A total of 133 patients were randomized, with 66 patients in the combination arm and 67 in the monotherapy arm. A variety of sarcoma histologic subtypes were included in this study including, but not limited to, leiomyosarcoma (36%), undifferentiated pleomorphic sarcoma (15%), liposarcoma (12%), and angiosarcoma (6%).

The primary endpoint of median PFS was found to be 6.6 months (95% confidence interval [CI] = 4.1–8.3) in the combination arm and 4.1 months (95% CI = 2.8–5.4) in the doxorubicin monotherapy arm. This met the protocol-defined significance level of 0.1999 for the final PFS analysis (stratified HR, 0.672; 95% CI = 0.442–1.021; *p* = .0615). The combination arm and doxorubicin monotherapy arm demonstrated objective response rates of 18.2% and 11.9%, respectively. Median overall survival in patients receiving olaratumab plus doxorubicin was 26.5 months (95% CI = 20.9–31.7) and was 14.7 months (95% CI = 9.2–17.1) with doxorubicin alone. The 11.8-month difference observed in median overall survival was found to be statistically significant (stratified HR, 0.46; 95% CI = 0.30–0.71, *p* = .0003). Some common adverse events experienced in the combination group included nausea, mucositis, neutropenia, and infusion-related reactions (IRRs). The authors concluded that the combination of olaratumab and doxorubicin met its predefined endpoint of improvement in PFS as well as significant improvement in overall survival as compared to doxorubicin monotherapy in patients with advanced STS.

## ADVERSE DRUG REACTIONS

The most common (occurring in ≥ 20% of patients) treatment-emergent adverse events with the combination of olaratumab and doxorubicin include nausea, fatigue, musculoskeletal pain, neutropenia, diarrhea, and mucositis (Eli Lilly and Company, 2017; Table 1). In the study by Tap and colleagues (2016), doxorubicin-associated adverse events such as neutropenia, mucositis, nausea, vomiting, and diarrhea were more commonly observed with the combination than doxorubicin monotherapy. Although more occurrences of grade 3 or higher neutropenia were observed in the combination of olaratumab and doxorubicin than with doxorubicin alone, the combination did not lead to an increase in febrile neutropenia, hospitalizations, treatment discontinuations, or deaths. It is recommended to dose reduce olaratumab to 12 mg/kg for patients who experience an infection or grade 4 neutropenia for longer than 1 week while on olaratumab (Eli Lilly and Company, 2017; Table 2).

**Table 1:**
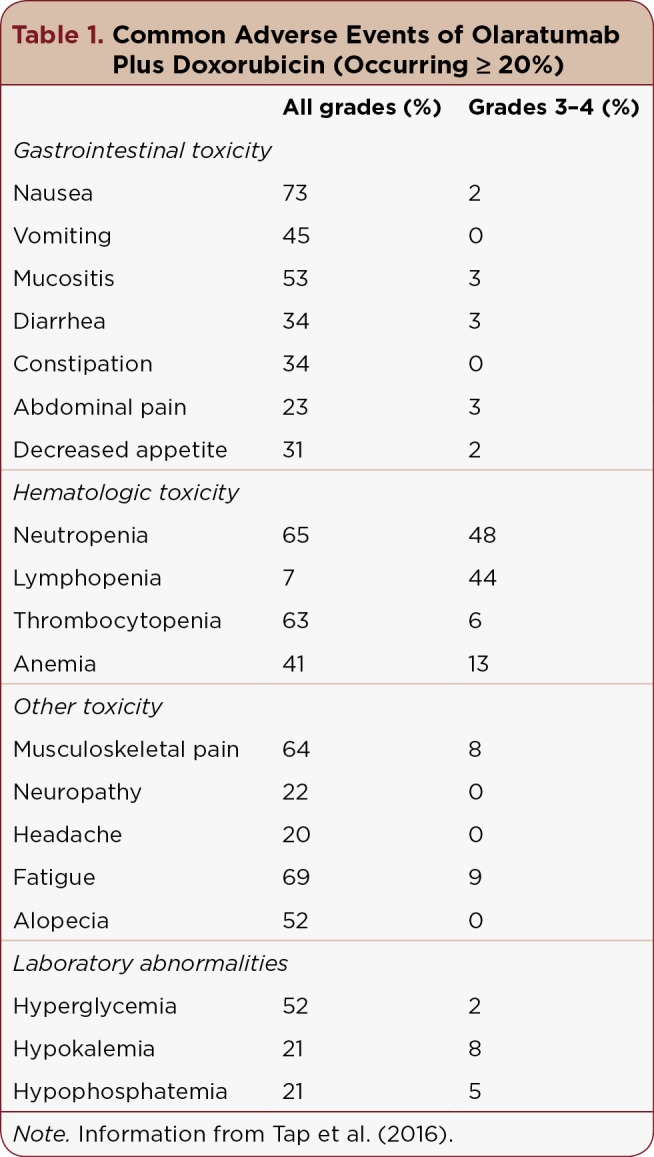
Common Adverse Events of Olaratumab Plus Doxorubicin (Occurring ≥ 20%)

**Table 2:**
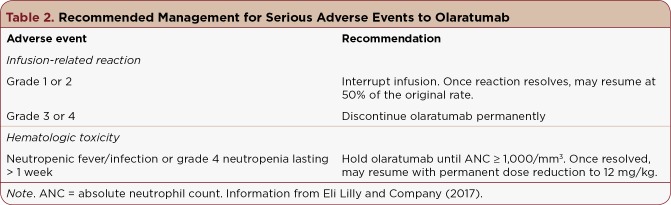
Recommended Management for Serious Adverse Events to Olaratumab

Olaratumab has been found to be associated with IRRs. An IRR of any grade may occur in up to 14% of patients receiving olaratumab. Approximately 3% of patients may experience a severe grade 3 to 4 IRR (Tap et al., 2016).

## DOSING AND ADMINISTRATION

The recommended dose of olaratumab is 15 mg/kg intravenously over 60 minutes on days 1 and 8 of a 21-day cycle (Eli Lilly and Company, 2017). The first 8 cycles are intended to be administered concomitantly with doxorubicin; upon completion of doxorubicin, olaratumab may be continued as maintenance therapy until disease progression or intolerable toxicity. There are no standard dose adjustments recommended for patients with mild to moderate renal or hepatic impairment. There are no data for dosing in patients with creatinine clearance < 30 mL/min or severe hepatic impairment; there are no recommended dose adjustments in this patient population.

## IMPLICATIONS FOR THE ADVANCED PRACTITIONER

Olaratumab offers the advanced practitioner a new option in the management of advanced STS for patients who are eligible to receive doxorubicin and whose disease is not amenable to curative resection or radiotherapy. Although several anthracycline-based combination chemotherapy regimens have been investigated in the treatment of STS, many of these regimens have not demonstrated improvements in overall survival despite having produced improvements in response rates (Bramwell, Anderson, & Charette, 2003; Judson et al., 2014, Santoro et al., 1995). Olaratumab, when given in combination with doxorubicin, exhibited prolonged overall survival, representing one of the only combination regimens in this setting to produce a survival benefit.

As previously discussed, olaratumab has been found to be associated with IRRs; advanced practitioners can be critical in the prevention and management of this adverse event. Most IRRs will occur with either the first or second cycle (Eli Lilly and Company, 2017). Advanced practitioners should monitor patients receiving olaratumab for flushing, dyspnea, hypotension, fever, and chills during the infusion. It is recommended to premedicate patients with diphenhydramine 25 to 50 mg IV and dexamethasone 10 to 20 mg IV prior to initiation of their first infusion. In the event of a grade 1 or 2 IRR, it is recommended to interrupt the infusion, and once the IRR has resolved, to reduce the rate by 50%. In the event of a grade 3 or higher IRR, it is recommended to permanently discontinue olaratumab.

Although the addition of olaratumab to doxorubicin has demonstrated an improvement in overall survival, the use of olaratumab has the potential to greatly increase the cost of treating STS. The average wholesale price for a 190 mg and 500 mg vial are $1,076.16 and $2,832.00, respectively (UpToDate, 2017). Also, with the combination of olaratumab and doxorubicin, the intent of administering doxorubicin is for 8 cycles, which would result in a cumulative doxorubicin dose of 600 mg/m². It is recommended to add dexrazoxane 750 mg/m² to prevent cardiotoxicity secondary to doxorubicin for cycles 5 to 8 of the treatment regimen. Although this does not directly affect olaratumab, the addition of dexrazoxane to the olaratumab/doxorubicin regimen can add further drug-related costs to an already costly antineoplastic regimen. Additionally, with the risk of cardiotoxicity with doxorubicin as companion therapy with olaratumab, a baseline echocardiogram or multigated acquisition scan should be performed to evaluate left ventricular ejection fraction. Routine surveillance imaging of cardiac function during the doxorubicin phase of treatment can be offered to patients at an increased risk for cardiac dysfunction (Armenian et al., 2017).

## ONGOING TRIALS

Olaratumab in combination with doxorubicin is currently being evaluated in a confirmatory phase III trial, the ANNOUNCE trial. Also, olaratumab is being evaluated in combination with gemcitabine and docetaxel in a phase Ib/II trial; this is a potentially very beneficial future direction for this therapeutic modality as it may help to expand the therapeutic potential of olaratumab to beyond to those who are unable to tolerate an anthracycline.

## CONCLUSION

Olaratumab is a novel anti-PDGFR-α monoclonal antibody indicated for the treatment of STS in combination with doxorubicin in patients with STS not amenable to curative surgery or radiotherapy. Being one of the only drugs in this setting to be combined with doxorubicin and demonstrate an overall survival benefit, olaratumab represents a promising addition to the armamentarium of therapies advanced practitioners have to treat STS.
